# Enhancing inclusion of neurodivergent participants in medical research: an intersectional inclusion-by-design approach

**DOI:** 10.3389/fmed.2026.1798656

**Published:** 2026-05-29

**Authors:** Waseem Jerjes, Ashley Arif, Azeem Majeed

**Affiliations:** 1 Faculty of Medicine, Imperial College London, London, United Kingdom; 2 Neurodiversity Advocate, London, United Kingdom

**Keywords:** health equity, inclusion-by-design, Intersectionality, neurodivergence, neurodiversity, participatory research, research accessibility

## 1 Introduction

Neurodiversity describes natural variation in how people think, communicate, learn, and process sensory and social information across the whole population. In this article, “neurodivergent” refers to people whose cognitive, sensory, attentional, communicative, or learning profiles diverge from dominant neurotypical expectations, including autistic people, people with attention deficit hyperactivity disorder (ADHD), dyslexia, dyspraxia and related neurodevelopmental profiles. As recognition grows, neurodivergent people are present across all clinical pathways and throughout the lifespan, far beyond specialist pediatric or disability services. Yet medical research still under-represents and under-supports neurodivergent people, treating accessibility as an exception rather than an expected feature of study design (1–5).

This is not merely a representation gap. It is a matter of scientific validity, ethics, and health equity. When design choices systematically filter out neurodivergent participants—through rigid recruitment, inaccessible consent, overstimulating settings, or narrow notions of “good communication”—findings become less generalizable. The evidence base can appear methodologically “clean” yet be clinically fragile: interventions may perform well in selected samples but translate poorly to real-world populations where neurodivergence influences engagement, adherence, adverse effects, and outcomes (5–10). The problem is amplified by the fact that neurodivergence may intersect with poverty, language barriers, ethnic minoritization, stigma, trauma histories and unequal access to diagnostic pathways, producing forms of exclusion that are not simply additive but mutually reinforcing (11). The outcome is predictable: those who most need research to reflect their experience are least likely to be counted by it (1, 4, 5).

The stakes are high because medical research does not only generate knowledge; it also sets norms about whose participation and outcomes count (6, 7). Research methods communicate who is expected to participate, whose accounts are treated as credible, and which outcomes are valued. When research processes assume a neurotypical default—rapid verbal exchange, comfort with ambiguity, tolerance of sensory overload, and the ability to “perform” calmness and coherence under time pressure—neurodivergent participants can be disadvantaged before a study begins. This is not a neutral inconvenience; it is a systematic source of bias. It shapes who is recruited, who stays, what data are collected, and how results are interpreted. Even when studies “include” neurodivergent participants, inclusion can be superficial if the environment and procedures remain misaligned with participant needs, resulting in rushed consent, masked distress, incomplete responses, or early withdrawal.

Contemporary evidence also makes clear that neurodivergent communities want research to improve lives and systems rather than simply describe deficits. Community perspectives repeatedly emphasize that research can be powerful when it attends to real-world priorities, respects lived experience, and shares decision-making power (3, 8, 9). Interdisciplinary scholarship also highlights tensions that emerge when neurodiversity is reduced to binaries (diagnosis vs. trait, impairment vs. difference, biology vs. context). It argues for research cultures that can hold these tensions without resorting to exclusionary simplifications (4, 10). These insights converge on a single practical conclusion: inclusion is not an optional “nice to have,” and it is not achieved by adding a paragraph on accessibility at the end of a protocol. Inclusion is a design discipline that should be planned, resourced, evaluated, and reported with the same seriousness as recruitment targets or statistical power.

This Opinion argues for an intersectional “inclusion-by-design” standard for medical research involving neurodivergent participants, positioning neurodivergence within the wider problem of who remains excluded from clinical research, why they are excluded, and how research systems can be redesigned. Such a standard requires changes in communication practices, sensory and environmental planning, consent processes, governance and ethics approaches, researcher capability, recruitment partnerships, ethnicity reporting, and funding priorities. It also requires humility: the assumption that traditional methods are inherently neutral must be replaced with the recognition that methods embed values and can unintentionally function as exclusion criteria. The goal is not to dilute rigor. The goal is to produce evidence that is both rigorous and relevant, and to do so through research processes that respect neurodivergent autonomy and expertise while recognizing intersecting barriers related to ethnicity, language, poverty and unequal diagnostic access.

## 2 Why neurodivergent people remain under-represented: barriers across the whole research pathway

**Figure 1:**
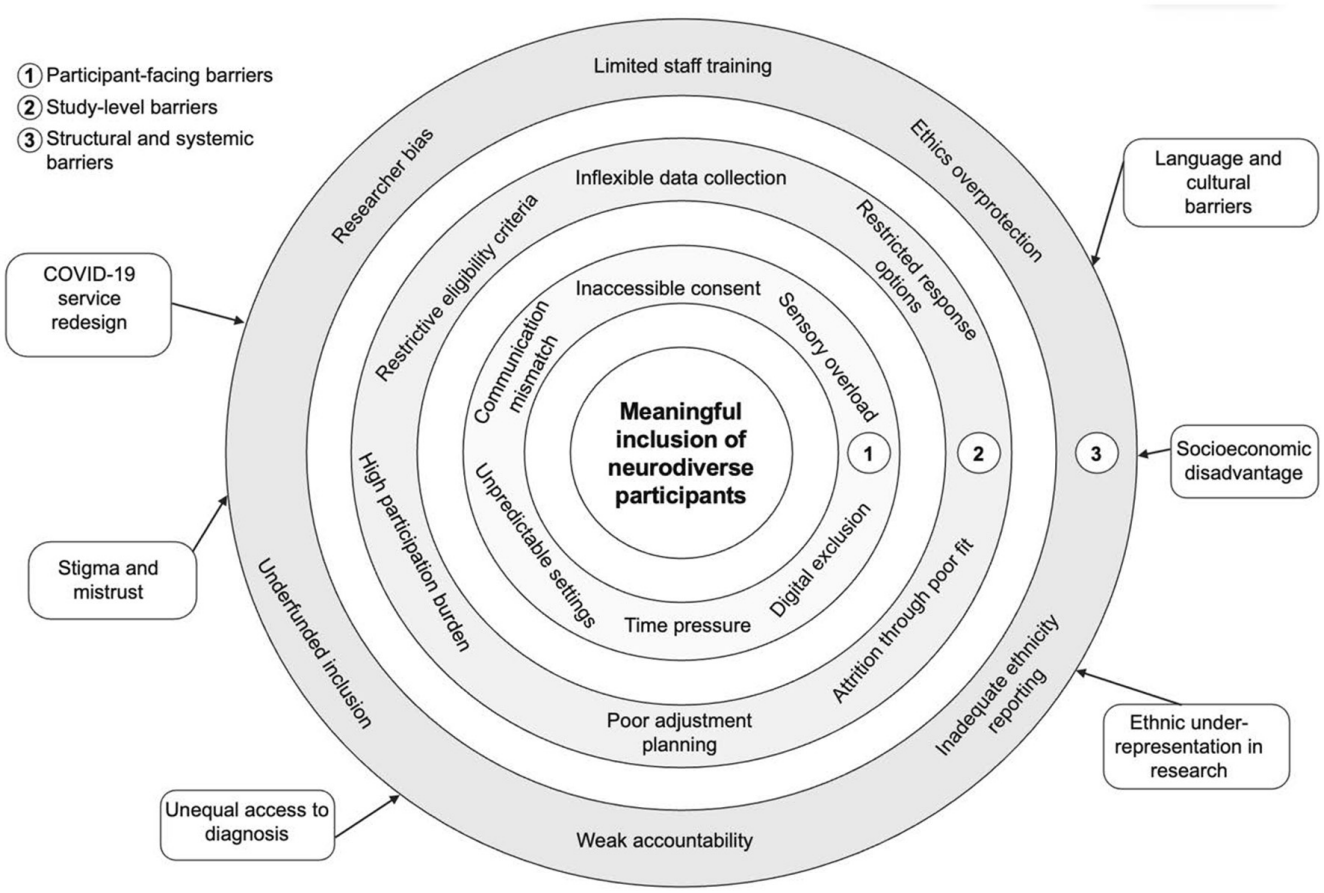
Circular model of intersecting barriers to inclusion of neurodivergent participants in medical research. The figure conceptualizes exclusion as a set of concentric layers rather than a single obstacle. The central circle represents meaningful inclusion of neurodivergent participants. The three surrounding layers represent participant-facing barriers, study-level barriers, and structural and systemic barriers that can limit inclusion across the research pathway. External factors such as stigma, socioeconomic disadvantage, unequal diagnostic access, language barriers and ethnic under-representation may further amplify these barriers across all layers.

Under-representation is often framed as a recruitment problem, when it is more accurately an accessibility problem: research systems are frequently less accessible to neurodivergent people. Exclusion can occur at every stage: recruitment materials, initial contact processes, eligibility rules, consent procedures, data collection formats, the sensory environment, staff attitudes, and follow-up expectations. Each stage can introduce friction that disproportionately affects neurodivergent participants and makes participation costly in ways that are rarely measured (14–17). Figure 1 illustrates how barriers to inclusion operate across interacting participant, study, and structural levels, with wider social and systemic factors further shaping who is able to participate in medical research.

Ethnicity deserves explicit attention along this pathway because exclusion is also a reporting problem. In autism intervention literature, race and ethnicity reporting remains inconsistent, and reported samples are often disproportionately White (11, 14). Broader inclusion work has emphasized culturally responsive recruitment and retention, family and community partnership, and greater transparency about who is reached and who remains missing (7, 8, 11). A paper focused on neurodiversity therefore becomes stronger, not weaker, when it acknowledges that inclusive research design must be both accessibility-sensitive and culturally responsive (11, 14).

Communication mismatch is among the most commonly cited barriers, but it is also often mislabeled as a participant deficit. The double empathy problem reframes communication breakdowns between autistic and non-autistic people as a two-way mismatch in understanding rather than a one-sided lack of social insight (12). Many study designs assume that reliable data require a single “standard” communication mode, typically spoken interaction at speed, in unfamiliar settings, with limited time to process. Neurodivergent participants may communicate differently, need longer to process questions, prefer written or asynchronous formats, use direct language, avoid eye contact, or find it difficult to produce immediate answers under observation. When research teams interpret these differences as unreliability or non-compliance, they inadvertently convert communication difference into exclusion. Even when research focuses on mental health experiences, the experiences themselves—and how well typical therapeutic or evaluative frameworks capture them—may differ for neurodivergent participants. This is particularly relevant where sensory overload, discrimination, or inaccessible communication environments shape lived experience (2, 18–23). If research tools assume a neurotypical model of distress and then conclude that neurodivergent people are “atypical,” the methodological problem has been mislabeled as a participant problem.

Environmental barriers can be equally decisive and should be approached through explicit design tools rather than *ad hoc* goodwill. The Autistic SPACE framework—Sensory needs, Predictability, Acceptance, Communication and Empathy, supported by physical, processing and emotional space—offers a practical way to translate accessibility into research planning (13). Clinical or university research settings often contain bright lights, unpredictable noise, crowded waiting areas, strong smells and uncertain scheduling, all of which can be considered in advance through SPACE-informed planning (13, 18, 19, 24–27). For participants with sensory sensitivities, these conditions can be exhausting or intolerable, and the energy cost of attending may not be visible to researchers. This invisibility matters because it affects who attends, how they perform during assessments, and whether they return. The same participant may provide rich, coherent data in a calm setting with adequate preparation, processing time and breaks, and minimal data in an overstimulating setting where self-regulation becomes the primary task. When this variation is not recognized, researchers may wrongly attribute differences in attendance, engagement or outcomes to the participant or intervention rather than to the context in which the data were collected.

Institutional caution can also become a barrier when it drifts into overprotection. Ethics committees may worry that neurodivergent participants cannot give valid consent or may be at higher risk of distress, and may therefore demand restrictive safeguards or exclude participants outright. The intention is to protect autonomy, yet the effect can be to deny autonomy by preventing participation. This is particularly problematic because it can result in avoidable exclusion despite the availability of proportionate safeguards (9, 20, 21, 28–33). When consent is treated as a one-off signature rather than a process that supports understanding over time, neurodivergent people may be harmed either by being excluded “for their own good” or by being included without adequate supports. Both outcomes represent failures of ethical imagination rather than inevitable realities.

Stigma and unconscious bias shape research in quieter ways. Some research teams assume that including neurodivergent participants will reduce data quality, increase attrition, or complicate timelines. This belief can influence decisions about who is approached, how information is presented, and how participant behavior is interpreted. It can also influence what outcomes are valued. When research continues to prioritize outcomes that are meaningful primarily to observers rather than to participants, such as appearing “normal” in behavior, neurodivergent people may reasonably distrust the purpose of participation. Community accounts frequently highlight that research is welcomed when it addresses lived priorities and system responsiveness, and resisted when it feels extractive or deficit-focused (3, 21, 22, 28).

Funding structures compound these barriers. Inclusive practices require time, training, adaptations and sometimes additional staff, including accessible participant materials, longer or split appointments, sensory-adapted rooms, remote participation infrastructure, interpreter or advocacy support, reimbursement of travel and carer time, and paid involvement for neurodivergent contributors. If funders do not explicitly value and resource these elements, inclusion becomes dependent on individual goodwill. For example, a protocol may state that written responses or breaks are permitted, but without funded staff time and flexible room booking, those adjustments may be unavailable in practice. This leads to variability: some teams do excellent inclusive work, while others deliver the minimal acceptable standard. Research that is underfunded for inclusion often externalizes costs onto participants and carers, particularly through long appointments, repeated visits, inflexible scheduling and unsupported digital access. This is not simply inconvenient; it can systematically exclude those with limited resources, precarious employment, caring responsibilities, or transport challenges. Survey evidence on healthcare access shows that adjustments are widely valued yet inconsistently available, reflecting a familiar pattern: needs are recognized, but systems do not reliably deliver (11, 23, 24, 29). Research settings risk reproducing the same pattern unless inclusion is treated as a measurable deliverable rather than a moral aspiration.

Finally, there is a conceptual barrier: neurodivergence is sometimes framed as a problem to be corrected rather than as a lens through which research environments can be redesigned. This matters because conceptual assumptions become operational decisions: a study that treats eye contact, rapid verbal fluency, sitting still or appearing calm as signs of engagement may misclassify neurodivergent participation. Similarly, a study that defines success as looking more “normal” may measure conformity rather than wellbeing, autonomy or meaningful participation. Interdisciplinary analysis has highlighted tensions in how neurodiversity is conceptualized and the opportunities this creates for more mature research cultures (4, 25, 30). If researchers assume that the participant must adapt to the research environment, exclusion follows. If researchers instead assume that the research environment must adapt to human diversity, inclusion becomes a technical, ethical and cultural task with measurable outputs.

## 3 Inclusion-by-design methods: improving accessibility without sacrificing rigor

A central methodological point is that rigor and accessibility are not opposites. In many cases, accessibility increases rigor because it improves the fidelity with which participants can express what they intend to communicate, reduces context-driven distress, and decreases systematic missingness. The practical challenge is to distinguish what must be standardized (construct, timing, scoring) from what can be flexible (response mode, pacing, environment, supports). Inclusion-by-design is not “anything goes.” It is planned flexibility with transparent reporting. Table 1 summarizes a practical inclusion-by-design framework.

**Table 1:** Inclusion-by-design framework for neurodivergent participation.

Domain	Design intention	Minimum deliverable to specify upfront	What to record and report
Communication	Reduce measurement error created by time pressure, social performance demands and one-way assumptions about communication	At least two response modes (for example, spoken and written), with planned switching rules	Mode used, switches, missingness patterns by mode
Predictability	Reduce anxiety driven by ambiguity and sequence uncertainty	Clear “what to expect” information and a defined session structure	Whether materials were provided; deviations from plan
Sensory environment	Minimize distress and dropout linked to avoidable sensory overload	A SPACE-informed low-sensory pathway, including attention to sensory needs, predictability, acceptance, communication, empathy and processing space	Adjustments requested and delivered; session tolerance
Consent accessibility	Protect autonomy through genuine understanding	Layered information and at least one alternative format	Comprehension checks; time taken; follow-up questions
Participant control	Prevent coerced endurance and support autonomy	Explicit right to pause, take breaks, or stop without penalty	Breaks taken; reasons for stopping when given
Staff capability	Reduce interpretive bias and improve respectful interaction	Neurodiversity-informed and neurodivergence-affirming training for participant-facing staff	Training completion; refresher schedule; incident learning
Co-production	Align study purpose and methods with lived priorities	Defined lived-experience role in design or governance	What changed as a result; ongoing feedback mechanisms
Digital flexibility	Increase access while avoiding new inequities	Supported hybrid pathway rather than “remote-only”	Technical barriers; digital exclusion mitigations
Retention focus	Treat dropout as a quality signal, not just a statistic	Pre-planned approach to reduce burden and follow-up friction	Attrition by subgroup and by participation pathway
Transparency	Make inclusion learnable and scalable	Standard reporting of adjustments and response modes	Publicly accessible method description and retention outcomes

First, communication should be treated as a design variable rather than an assumption. Where possible, studies should offer more than one mode of participation, such as written responses, asynchronous options, or structured interview formats that allow pauses and clarifications. This reduces measurement error associated with time pressure and high social-performance demands, and avoids conflating communication style with insight or capacity. Evidence that experiences of anxiety and therapy can diverge from neurotypical models, partly due to sensory and communication contexts, demonstrates why method flexibility is not cosmetic but epistemically important (2, 26, 27, 30).

Second, predictability should be treated as a core accessibility feature. Many neurodivergent participants benefit from knowing what will happen, in what order, for how long, and with what sensory demands. Providing clear “what to expect” materials, appointment structure, options for breaks, and explicit consent to pause or stop can reduce stress and improve data quality. Co-production work has demonstrated the value of agreed ground rules and structured systems that support collaboration and reduce ambiguity (20, 28, 31). Research teams can adapt this principle by establishing participant-facing ground rules that explicitly legitimize needs such as time to process, reduced eye contact, or written follow-up, without interpreting these as disengagement.

Third, the sensory environment should be planned, not improvised. Research often treats the setting as fixed, yet small changes can have disproportionate benefits: quiet waiting areas, reduced lighting intensity, minimized background noise, and predictable scheduling that avoids long uncertain delays. Where the physical environment cannot be reliably adjusted, offering remote or hybrid options can reduce sensory burden. The rapid expansion of remote methods during the pandemic demonstrated that some barriers can be reduced through telehealth, while also highlighting that remote delivery can introduce new barriers, including increased rigidity, technology challenges, and inequities related to digital access (15, 16). The key point is that choice matters, and that options must be supported rather than merely offered.

The COVID-19 period sharpened this lesson. Rapid shifts to remote and hybrid methods improved access for some autistic adults, yet they also revealed new barriers linked to digital exclusion, system rigidity, and unequal recruitment. Pandemic-era clinical-trial literature showed that diverse participation did not improve automatically under emergency conditions, with continuing under-representation or inconsistent classification of minoritized ethnic groups. Remote methods should therefore be framed as tools for optional flexibility, not as inherently inclusive solutions (34–37).

Fourth, eligibility criteria and outcome measures should be examined for unintended exclusion. Neurodivergent participants may be excluded by rules that require certain styles of communication or compliance that are not essential to the scientific question. For example, requiring participants to complete long questionnaires in one sitting, attend at fixed times without flexibility, or tolerate sensory discomfort for non-essential procedures can reduce participation and distort samples. Where feasible, micro-adjustments such as shorter sessions, splitting assessments, and allowing support persons can preserve the core measurement while reducing participant burden. These are straightforward methodological decisions with clear impacts on recruitment and retention.

Fifth, inclusion requires attention to the full participation pathway, not only the initial encounter. Attrition is often treated as a statistical nuisance rather than as a signal of poor accessibility. If neurodivergent participants disproportionately withdraw, it suggests that study burden, communication mismatch, or environment is misaligned. Rather than simply increasing recruitment numbers to compensate, teams should treat attrition patterns as quality indicators. Open and transparent research cultures can strengthen this work: combining participatory approaches with open practices can improve trust, accountability, and reproducibility, while making accessibility lessons easier to document and share (10, 21, 29). Transparent reporting of adaptations, response modes, and retention patterns should become a norm rather than an exception.

Sixth, inclusion is strengthened when communities have a role in shaping research priorities and methods. Priority-setting work indicates that stakeholders repeatedly emphasize research that leads to real-world improvements and meaningful outcomes across the lifespan (21, 29). Community perspectives also emphasize that research can be positive and transformative when it shifts away from narrow deficit framing and listens to lived experience (3). This implies that inclusion cannot be separated from purpose: people are more likely to participate when they recognize themselves in the research question and believe the outputs will matter. Participatory research methods have been described as flexible yet sometimes confusing in practice, and those observations are instructive: inclusion improves when teams are explicit about the form of involvement, the boundaries of decision-making, and the resources available (19, 32).

Seventh, training and culture change are methodological necessities, not optional extras. Researchers cannot deliver inclusive methods if they have not been trained to recognize neurodivergent communication, to respond without judgement, and to distinguish distress caused by sensory mismatch from distress caused by the content of questions. Training is also needed to avoid interpretive bias. For example, if a participant appears “flat” or avoids eye contact, this should not automatically be coded as disengagement. Without training, research teams can inadvertently create data artifacts through misinterpretation of behavior. A growing body of work across different practice settings emphasizes the need to align methods with a neurodiversity-affirming stance that centers dignity and autonomy (6, 15, 31). In research terms, this means designing procedures that do not treat neurodivergent traits as noise to be suppressed, but as context that should be respected and measured appropriately.

## 4 Governance, ethics, and infrastructure: building systems that make inclusion routine

Inclusion will remain uneven unless it is anchored in governance structures that create accountability and require specific, auditable actions. Ethics processes, funder expectations, organizational policies, and journal reporting norms can either reinforce exclusion or normalize accessibility. The task is to treat reasonable adjustments in research as core safeguards that protect autonomy and improve validity, rather than discretionary “extras.”

Consent is the clearest place where ethics and accessibility must be integrated. Traditional consent documents are frequently long, complex, and oriented toward institutional protection rather than participant understanding. For neurodivergent participants—particularly those with processing differences, anxiety under time pressure, or communication differences—this can undermine meaningful informed consent. Recent guidance on accessible consent materials and procedures demonstrates that the solution is not to lower standards, but to redesign materials and processes so that understanding is more likely: layered information, clearer language, alternative formats, explicit accommodation of processing and communication differences, and opportunities to revisit decisions over time (9, 19, 28). Importantly, accessible consent should be framed as good practice for everyone; neurodivergent-focused accessibility improvements often enhance comprehension across the whole participant population. An ethics application could therefore include a short neurodivergence access plan specifying available communication modes, layered information, comprehension checks, break options, supporter involvement, sensory adjustments and procedures for documenting preferences without pathologizing the participant.

Ethics committees and governance bodies should shift from “avoiding risk by excluding complexity” to “reducing risk through better design,” meaning that foreseeable barriers should be anticipated and addressed within the protocol rather than managed through exclusion. This is particularly important because excluding neurodivergent people can itself be ethically problematic, as it denies the benefits of participation and perpetuates evidence gaps. The correct response to uncertainty about consent capacity is not blanket exclusion, but a proportionate, supportive consent process using plain-language summaries, teach-back methods, additional processing time, supporter involvement where requested, and repeat opportunities to confirm consent over time. This links consent capacity to design: the ethical question is not whether a participant can navigate a standard form under standard conditions, but whether the consent process has been designed to support understanding.

Co-production guidance provides a complementary governance lens: inclusion is strengthened when power and responsibility are shared from early design through dissemination, and when relationships, reciprocity, and resourcing are explicit (8). This is not merely a philosophical point. It affects practical decisions such as compensation, scheduling, and feedback loops. If neurodivergent participants contribute expertise, they should be compensated appropriately, and research timelines should include time for genuine involvement. Public involvement guidance also emphasizes that involvement must be planned and supported, not assumed to happen spontaneously (7, 27, 31). These principles apply directly to neurodivergent inclusion: if research expects neurodivergent people to participate, then the infrastructure must support their participation in a respectful and sustainable way.

Infrastructure includes staffing models. Inclusion cannot be delivered by one enthusiastic team member with niche expertise. It requires team-wide competence and clear processes. This includes consistent methods for documenting participant preferences, providing adjustments, and ensuring continuity across visits. It also includes the ability to offer hybrid participation where possible. Evidence from telehealth work shows both the promise and limitations of remote delivery, and highlights that flexibility and individual fit matter more than any single technology (15, 16, 29, 31). A governance implication is that studies should plan remote participation pathways with the same seriousness as in-person pathways, including troubleshooting, privacy, and safeguarding.

Funding is a structural determinant of inclusion. When funders treat inclusion as an optional “impact” add-on, teams will cut it first when budgets tighten. When funders require explicit inclusion plans and budget lines—training, accessible materials, participant support, and involvement costs—inclusion becomes deliverable rather than rhetorical. A practical example of this broader shift is the King's Model for minority ethnic research participant recruitment. Developed during efforts to improve racially diverse involvement in clinical research, including in the COVID-19 context, it combines local infrastructure, community partnership, tailored outreach, and long-term implementation planning rather than relying on generic recruitment appeals. Its relevance to neurodivergent inclusion is direct: sustainable participation depends on relationships, trust, and system design, not on asking excluded communities to overcome institutional barriers unaided (38). Evidence on research priorities also suggests that aligning research questions with stakeholder priorities matters for legitimacy and participation (21). Funders therefore shape inclusion not only through budgets but through what questions and outcomes are valued.

Accountability also depends on reporting standards. Community involvement reporting requirements and open research practices are increasingly discussed as ways to improve transparency, reproducibility, and trust (10, 19, 32). The same logic should apply to accessibility. Research papers should be expected to report, in a standardized way, what adjustments were available, what response modes were offered, how consent was supported, and whether retention differed across groups. This does not require excessive length; it requires disciplined reporting. Without such transparency, it is impossible to learn systematically from what works, and inclusive practice remains artisanal rather than scalable.

Finally, inclusion must address the translational interface between research and care. Research participation is shaped by broader healthcare experiences. Evidence on barriers to healthcare access illustrates how communication mismatch, doubt and dismissal, and accumulated negative experiences can lead to avoidance and adverse outcomes (17, 27, 28). If neurodivergent participants approach research with prior experiences of not being believed or being overwhelmed by systems, recruitment messaging and researcher behavior must actively counter those expectations. Trust is not generated by a consent form; it is generated by repeated signals that the participant's communication is valued, that adjustment requests will be respected, and that participation will not require self-erasure. Primary care-focused work also suggests that service design matters, and that participants often support structured approaches when they include opportunities to communicate preferences and needed adjustments (18, 29, 31). Research can learn from this: the more explicitly studies invite participants to state what helps, the more feasible and predictable participation becomes.

In summary, governance and infrastructure determine whether inclusion is sporadic or routine; accessibility must be built into consent, environment, staffing, funding, and reporting.

## 5 Way forward: making inclusion-by-design operational

Neurodivergent inclusion should be treated as a scientific and ethical standard of modern medical research, and the aim of this Opinion is to make that standard operational rather than simply aspirational. The evidence base already provides a coherent direction: communities want research that improves lives, research cultures benefit from participatory approaches, accessible consent can be designed without compromising autonomy, and flexible methods can reduce barriers while improving data fidelity (3, 9, 10, 19, 21, 28, 32). The remaining challenge is operational: translating these insights into routine protocol design, ethics review, funding assessment, researcher training and journal reporting.

That operational challenge should now be understood in intersectional terms. A neurodiversity agenda that ignores ethnicity, language, poverty, gender, age, migration status or diagnostic inequity will still produce selectively inclusive research. The strongest future studies will therefore treat neurodivergent inclusion and representative recruitment as linked responsibilities, using the same seriousness applied to sample size, retention, and outcome selection (11, 13, 36, 39). In our own research and educational work, we are applying this argument by treating accessibility as an early design question rather than an end-stage adjustment. This includes using plain-language information, offering written as well as spoken communication where feasible, allowing processing time and breaks, considering sensory and digital access when planning data collection, and involving advocacy or lived-experience perspectives in the framing of research questions.

A key risk is that inclusion becomes a box-ticking exercise. This is best addressed by embedding practical checklists within a broader culture of accountability, shared power and transparent reporting. Table 1 is not intended to substitute for thoughtful design. It is intended to prevent predictable omissions and to encourage teams to make inclusion decisions early rather than late. The deeper work is cultural: moving from a deficit framing that asks how neurodivergent people can be made to fit research, to a design framing that asks how research can be made to fit human diversity.

Another risk is homogenization. Neurodivergence is not a single profile, and adjustments are not one-size-fits-all. What helps one person may hinder another. This is why choice architecture matters. Offering multiple modes of participation, providing predictable structure while allowing flexibility, and treating adjustments as normal rather than exceptional allows individual fit without methodological chaos. Planned flexibility is not a threat to rigor. Unplanned variability is. The goal is to specify in advance what can vary, why it can vary, and how it will be documented.

Inclusion also has interpretive consequences. When neurodivergent people are excluded, the field risks building theories and interventions that interpret neurotypical behavior as the benchmark and neurodivergent behavior as deviation. When neurodivergent people are included meaningfully, research questions often change: from “why is the person different?” to “how do contexts, systems, and expectations produce disability and distress?”. Interdisciplinary work has highlighted the value of holding multiple frames—biological, psychological, social—without forcing simplistic binaries (4, 23, 28). Community perspectives reinforce that research becomes more useful when it attends to context and systems rather than narrowing its gaze to deficits (3, 31).

There is also a pragmatic, health-system argument. Research that excludes neurodivergent participants can inadvertently waste resources by producing interventions that are less implementable in routine care. The gulf between trial conditions and real-world conditions is already a major challenge in translational medicine. Neurodiversity magnifies this gulf when trials assume capacities and tolerances that are not evenly distributed across populations. Inclusion-by-design therefore improves not only fairness but efficiency: interventions are more likely to work when they are tested in populations that resemble those who will receive them.

The strongest lever for change is to make inclusion measurable and reportable. Funders can require costed inclusion plans, evidence of community involvement and explicit budgets for accessibility work (7, 8, 18, 29). Ethics committees can evaluate consent accessibility, adjustment planning and neurodivergence access plans as safeguards rather than as optional extras (9). Research teams can treat attrition patterns as quality signals and can report accessibility choices, response modes, consent supports and retention patterns transparently, aligning with broader movements toward openness and accountability (10). Training can be made a core competency for research staff, supporting respectful communication and reducing interpretive bias (6, 27). The resulting ecosystem would not rely on exceptional individuals; it would rely on normalized standards.

Ultimately, inclusion is a statement about whose knowledge counts. Neurodivergent participants are not merely subjects of research; they are experts in their own experience and can illuminate the mismatch between systems and people. When research is designed to include them, the benefits extend beyond any single study. The field gains better questions, better methods, and better translation. The public gains evidence that reflects the diversity of real lives.

Inclusion is both an ethical mandate and a scientific opportunity. The next stage of medical research should assume neurodivergent inclusion as the default and judge study quality partly by whether participation was made genuinely possible for those whose lives the evidence claims to represent. If research is serious about equity, relevance and trust, inclusion-by-design should be treated as a routine intersectional standard: planned in protocols, resourced in budgets, evaluated by ethics committees, reported by journals, and developed with explicit attention to ethnicity, accessibility, co-production and transparent demographic reporting.

## 6 Positionality and lived-experience involvement

The author team brings clinical, academic, advocacy and close lived-experience perspectives related to neurodivergence, and includes at least one author who is a neurodivergent. A neurodiversity advocate contributed as a co-author to the framing, terminology and practical recommendations in this article. We recognize that no author team can represent the full heterogeneity of neurodivergent communities, particularly across diagnosis, ethnicity, language, age, gender, socioeconomic position and support needs. The recommendations are therefore offered as a framework to be adapted, tested and governed with neurodivergent participants and community partners in future empirical research.
